# Cramps in the Leg

**Published:** 1888-11

**Authors:** 


### CRAMPS IN THE LEG.

Many persons of both sexes are greatly troubled with cramps in one or both their legs. It comes on suddenly and is very severe. Most people jump out of bed (it nearly always comes on either just after going to bed, or while undressing) and ask some one to rub the leg. I have known it to last for hours, till in despair they would send for the family physician ; and even then it would be hours before the spasm would let up.

There is nothing easier than to make the spasm let go its hold, and it can be accomplished without sending for a doctor, who may be tired and in need of a good night's rest. When I have a patient who is subject to cramp I always advise him to provide himself with a good strong cord. A long garter will do if nothing else is handy. When the cramp comes on, take the cord, wind it around the leg over the place that is cramped, and take an end in each hand and give it a sharp pull,—one that will hurt a little. Instantly the cramp will let up and the sufferer can go to bed assured it will not come on again that night.—DR. ST. CLAIR.

